# Superior Fluorescent Nanoemulsion Illuminates Hepatocellular Carcinoma for Surgical Navigation

**DOI:** 10.3389/fbioe.2022.890668

**Published:** 2022-04-25

**Authors:** Jing Zhu, Chengchao Chu, Dongsheng Li, Yang Zhang, Yi Cheng, Huirong Lin, Xiaoyong Wang, Junxian Liu, Xin Pang, Jingliang Cheng, Gang Liu

**Affiliations:** ^1^ Department of Magnetic Resonance Imaging, The First Affiliated Hospital of Zhengzhou University, Zhengzhou, China; ^2^ Center for Molecular Imaging and Translational Medicine, School of Public Health, Xiamen University, Xiamen, China

**Keywords:** Hepatocellular Carcinoma, fluorescence imaging, nanoemulsion, indocyanine green, surgical navigation

## Abstract

Hepatocellular carcinoma (HCC), the fifth most common cancer worldwide, poses a severe threat to public health. Intraoperative fluorescence imaging provides a golden opportunity for surgeons to visualize tumor-involved margins, thereby implementing precise HCC resection with minimal damage to normal tissues. Here, a novel-acting contrast agent, which facilely bridges indocyanine green (ICG) and lipiodol using self-emulsifying nanotechnology, was developed for optical surgical navigation. Compared to clinically available ICG probe, our prepared nanoemulsion showed obviously red-shifted optical absorption and enhanced fluorescence intensity. Further benefiting from the shielding effect of lipiodol, the fluorescence stability and anti-photobleaching ability of nanoemulsion were highly improved, indicating a great capacity for long-lasting *in vivo* intraoperative imaging. Under the fluorescence guidance of nanoemulsion, the tumor tissues were clearly delineated with a signal-to-noise ratio above 5-fold, and then underwent a complete surgical resection from orthotopic HCC-bearing mice. Such superior fluorescence performances, ultrahigh tumor-to-liver contrast, as well as great bio-safety, warrants the great translational potential of nanoemulsion in precise HCC imaging and intraoperative navigation.

## Introduction

Surgical resection remains the paramount treatment option for liver tumor, such as hepatocellular carcinoma (HCC), and metastases of gastroenterological cancers (Siegel et al., 2019). Successful oncologic surgery is predicated on accurate visualization of the neoplastic tissue so as to precise removal with minimal damage to normal tissues (Hasegawa and Kokudo, 2009). However, in clinical practice, surgeons usually cannot discriminate the intraparenchymal tumor accurately only by visual inspection and tactile feedback. Their failure to pinpoint the tumor margins results in positive surgical margins, which are correlated with poor prognosis, locoregional recurrence and low patient survival. Promisingly, intraoperative fluorescence imaging provides a golden opportunity for surgery guided due to its high contrast, low cost, real-time feedback, and good availability of imaging whole organs with the potential to zoom in from macroscopic scale to microcosmic structures (Verbeek et al., 2012; Gao et al., 2018; Ren et al., 2020). More importantly, it is easily integrated into the workflow of intraoperative surgery and endoscopy, significantly augmenting the visualization ability of surgeons in identifying tumor-involved margins (Mochida et al., 2018; Wang et al., 2018). As the only near-infrared (NIR) fluorophore approved for surgery intraoperative guidance, indocyanine green (ICG), which emits at ∼800 nm, effectively minimizes photo scattering, absorption and tissue autofluorescence while maximizing tissue penetration depths to several centimeters (Zhang et al., 2017). With the aid of ICG-guided intraoperative imaging, a fluorescent edge of the cancerous tissue can be observed during surgery for margin delineation between cancer and adjacent healthy tissue (Gotoh et al., 2009; Hirche et al., 2010; Xu et al., 2020). Although it holds great promise in improving intraoperative decision-making, this untargeted small-molecule dye still suffers from non-specific binding and usually high background noise. Moreover, fast clearance from tumor site further limits the time window of ICG for surgical applications, and the severe photobleaching and degradation issues also frequently reduce its detectability or observation time (Mindt et al., 2018; Sakka, 2018; Chen et al., 2020a). Consequently, a more efficacious fluorophore that allows tumor-targeted imaging, long-lasting HCC retention, and stable fluorescence signal, is urgently desired.

In the fluorescence-guided surgical navigation, the signal to noise ratio (SNR) of ICG relies on its accumulation and retention in liver cancerous tissues (Ai et al., 2018). Lipiodol (Andre Guerbet, Aulnay-sous-Bois, France), an iodized poppyseed oil, has been most commonly used as an embolic material for transcatheter arterial chemoembolization against HCC in clinic (Lencioni et al., 2016; Chen et al., 2020b). It demonstrates a preferential uptake by hypervascular tumor compared to surrounding liver tissue, which can be used as a vehicle for the targeted delivery of cytotoxic drug or contrast agent (Kawaoka et al., 2009; Jeon et al., 2016; Gruber-Rouh et al., 2018). When combination with these functional agents, the lipiodol is expected to act as a shelter to protect internal agents from dilution and degradation by adverse external environment. Moreover, the high viscosity of lipiodol makes it prone to deposit in the anomalous vessel of HCC, which is a great help for internal agents resist blood flow clearance (Cheng et al., 2020). Further benefiting from the reduced molecular movement, the functional agents inside highly viscous lipiodol expectantly possess increased stability. Some potential problems such as fluorescence quenching of ICG is therefore promisingly relieved (Chen et al., 2020c). However, the task to integrate lipiodol with ICG using a safe and facile platform remains a major challenge. Ideally, the functional agents should be “loaded” inside the droplets of lipiodol (water-in-oil emulsion) for an optimal tumor uptake (De Baere et al., 1996). Stabilization of the emulsion is also a key for lipiodol to serve as a vehicle to carry and localize the ICG inside the cancerous tissue. Thus, solutions for ICG-guided surgical navigation, in the long-run, require a multipronged cooperation.

**SCHEME 1:**
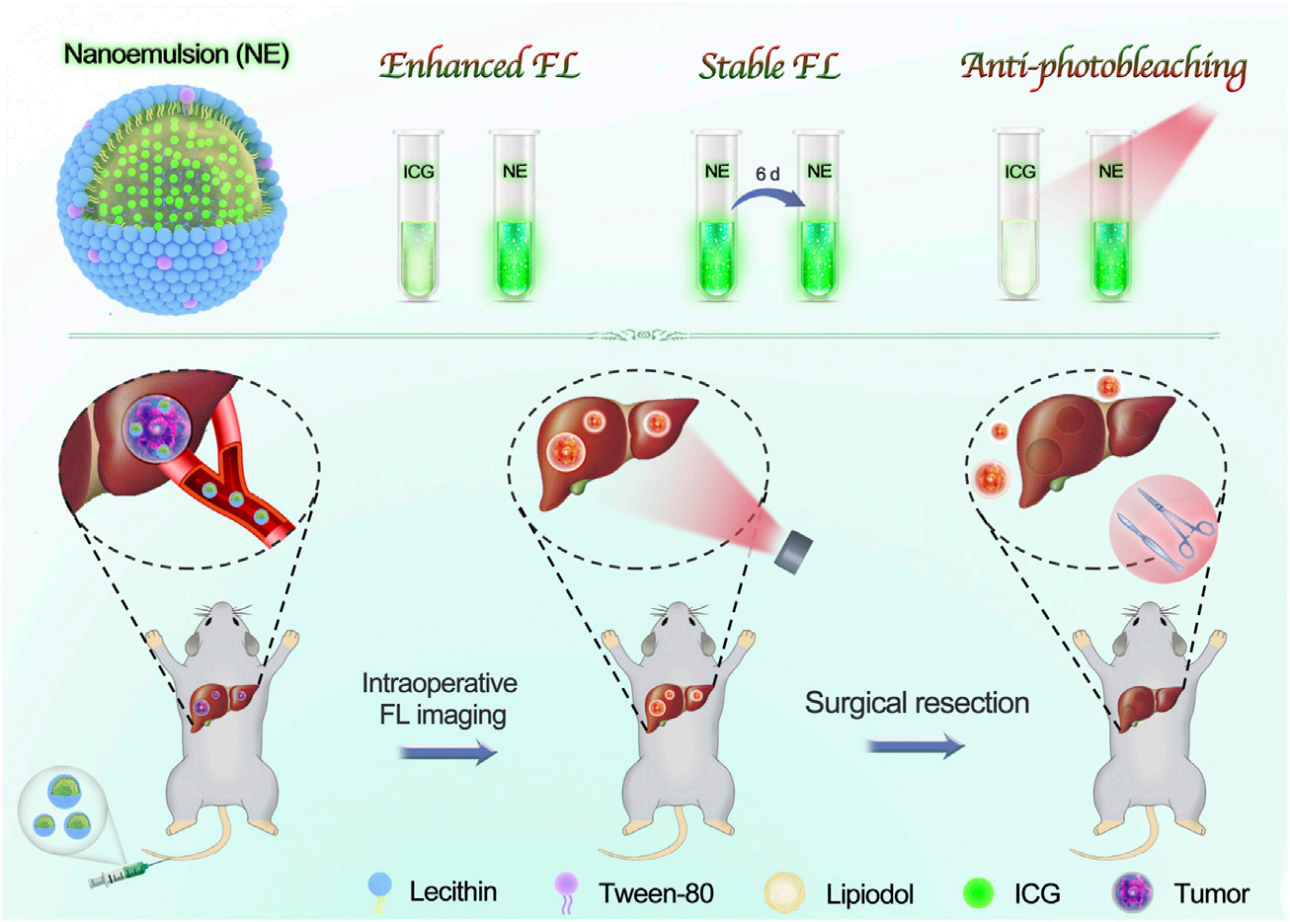
Scheme illustration of our developed nanoemulsion for FL imaging-guided surgical resection of HCC. The nanoemulsion was facilely prepared with clinically approved materials, including ICG, lipiodol, lecithin, and tween-80. By taking advantage of superior FL performances and ultrahigh tumor accumulation, the tumor tissues were clearly delineated, and then underwent a complete surgical resection from orthotopic HCC-bearing mice.

Nanotechnology has revolutionized medicine including fluorescence-guided intraoperative imaging (Ai et al., 2018). Unlike small molecular designs, the cooperative behaviors of nanosystems, which take advantage of well-developed material engineering and the enhanced permeability and retention (EPR) effect, can be exploited for the development of highly efficient nanoemulsion to amplify pathological signals, and further improve the accuracy of intraoperative imaging (Li et al., 2018; Li et al., 2019). Here, a facile route to bridge lipiodol and ICG was proposed using self-emulsifying nanotechnology **(** Scheme 1 **)**. As a proof-of-principle, Tween-80 and lecithin, the clinically available surfactants, were introduced to emulsify the mixture of lipiodol and ICG. Strikingly, the developed nanoemulsion obviously shifted excitation and emission peaks and produced enhanced fluorescence intensity compared with free ICG, which suggests superior potential for NIR intraoperative imaging. With the aid of shielding effect by lipiodol, the fluorescence stability and anti-photobleaching ability of nanoemulsion was greatly improved, indicating the capacity for real-time *in vivo* imaging continuously for long periods. Moreover, this nanoemulsion could successfully localize to neoplastic tissue with excellent tumor-to-normal contrast. Under the fluorescence guidance of ICG-lipiodol nanoemulsion, the tumor tissues from orthotopic HCC-bearing mice were clearly visualized, accurately delineated and then effectively resected.

## Experimental Section

### Materials

ICG, Lipoid and Tween-80 were obtained from Aladdin (China). 4′,6-Diamidino-2-phenylindole (DAPI) and 3-[4,5-dimethylthiazol-2-yl]-2,5-diphenyl tetrazolium bromide (MTT) were obtained from Sigma-Aldrich. Fetal bovine serum (FBS) and high glucose DMEM were purchased from Hyclone (United States). Penicillin and streptomycin were obtained from GIBCO BRL (United States).

### Preparation of Nanoemulsion

5 mg ICG was dissolved in 5 ml chloroform, and then mixed with 500 μL iodinated oil. The mixture was removed chloroform by rotary evaporation. 100 mg Lipoid was dissolved in 300 μL ethanol. 400 μL Tween-80 was dispersed in 20 ml distilled water and brought to a boil. Subsequently, the Lipoid solution and the mixture were added into the above-mentioned boiling liquid dropwise under continuous stirring (700 rpm). The preparation was concentrated to 10 ml. Finally, the crude emulsion was homogenized for 15 min using a sonicator tip (Sonics, United States) to form uniform nanoemulsion. The final nanoemulsion was obtained by using dialysis (30 KDa) and stored in dark at 4°C.

### Characterization of Nanoemulsion

The morphology of nanoemulsion was observed by transmission electron microscopy (TEM). The dynamic light scattering (DLS) and surface zeta potential of nanoemulsion were captured using a Nano Particle analyzer (SZ-100, Horiba Scientific). UV–vis–NIR absorbance spectra of ICG and nanoemulsion were recorded using a microplate reader (Thermo). Fluorescence emission spectrum of ICG and nanoemulsion were recorded using fluorescence spectrometer (LS55, Perkin Elmer), the excitation wavelength was 780 nm. The FL images of ICG and nanoemulsion (gradient concentration) were acquired using a IVIS Lumina imaging system (Caliper, United States) (Ex/Em = 740 nm/ICG). The fluorescence intensity of nanoemulsion and ICG solution (10 μg/ml) were measured after different laser irradiating time (808nm, 2 W/cm^2^) or at different storage time. The nanoemulsion with different iodine concentration were scanned by a micro-CT imaging system. The PA images of nanoemulsion were acquired using a preclinical PA imaging system (Endra Nexus 128, Ann Arbor, MI) under 808 nm laser. The stability of nanoemulsion in PBS solution and DMEM were evaluated at different time points by DLS.

### *In vitro* Cell Uptake and Cytotoxicity Study

HepG2 and normal hepatocytes (LO2) were purchased from the cell bank of the Chinese Academy of Sciences in Shanghai. Cells were cultured in DMEM containing with 10% (v/v) FBS and 100 µg/ml of penicillin/streptomycin at 37°C under 5% CO2. The HepG2 cells were seeded into 6-well microplates at a density of 5 × 10^5^ cells per plate, and incubated for 12 h. Then, the culture media was replaced by fresh media containing ICG or nanoemulsion (1 ml, at the equivalent ICG dose of 10 µg/ml in DMEM), respectively. After co-incubation for different time points, the cellular uptake was analyzed by fiow cytometry equipment. Furthermore, the CLSM was applied to study the cell uptake of nanoemulsion. Briefiy, HepG2 cells were seeded into a CLSM-specifc dish at a density of 1 × 10^5^ cells per dish, and incubated for 12 h for adherence. Then, the culture media were replaced by fresh media containing nanoemulsion or ICG (1 ml, at the equivalent ICG dose of 10 µg/ml in DMEM) and incubated for 12 h. For locating the cell, 10 µL of DAPI was added into each dish to stain the cell nuclei for 15 min followed by observation with CLSM.

The cytotoxicity of the ICG and nanoemulsion was assessed by 3-(4,5-dimethylthiazol-2-yl)-2,5-diphenyltetrazolium bromide (MTT) assay. The HepG2 and LO2 cells were seeded into 96-well microplates and cultured overnight. Then, the culture media were replaced by fresh media containing ICG and nanoemulsion with gradient concentrations of ICG and cultured for 24 h. Finally, the cell viability was detected by MTT assay.

### Animal Model Establishment

All animal experiments were performed according to the protocol approved by the Animal Care and Use Committee of Xiamen University, China. For the establishment of tumor model, the female Balb/c nude mice (5–6 weeks, 22 ± 2 g) were supplied by the Center for Experimental Animals, Xiamen University, China. Orthotopic liver tumor models were established by inoculation of suspended HepG2 cells (25 µl, 1 × 10^7^ cells/ml) at the left lobe of liver. The models were used for further experiments when the tumor grown up.

### Orthotopic Liver Tumor PA and Fluorescence Imaging

After the model of tumor-bearing mice was established, the mice were randomly divided into two groups: ICG and nanoemulsion. The dose of ICG (2 mg/kg) was administrated by intravenously injection in each group. PA signals in the liver and tumor were observed using Endra Nexus. The FL images were acquired before and at different time points (1, 3, 6, 9, 12, and 24 h) after administration using IVIS Lumina imaging system (Caliper, United States) (Ex/Em = 740 nm/ICG). At 24 h post injection, the mice were sacrificed and their major organs (heart, liver, spleen, lung, and kidney) and the tumor were harvested for *ex vivo* FL imaging. The fiuorescence signal intensity was quantified by analyzing the signals at the region of interests.

The mice were randomly divided into two groups, After i.v. administration of nanoemulsion and ICG (2 mg/kg), the mice were sacrificed at scheduled time (0, 1, 3, 6, 12, 24, and 36 h) and were taken for *in vitro* FL imaging. The fiuorescence signal intensity of liver and tumor were quantified, and the signal-to-noise ratio (SNR) of FL imaging was calculated:

SNR = FL intensityTumorFLintensityLiver

### Surgical Resection of Orthotopic Liver Tumor

Orthotopic liver tumor mice were performed surgical excision at 24 h with i.v. administration of nanoemulsion (2 mg/kg). Surgeons were asked to remove tumor tissues with maximum protection of the normal tissue based on real-time fluorescent surgical navigation. If tumor tissue remained, the surgeons continued the resection with FL imaging guidance until removing all tumor tissues. The resected tissues were also analyzed by H&E staining.

### *In vivo* Biocompatibility Assessment

Healthy BALB/c nude mice (20–22 g) were intravenously injected with PBS, ICG or nanoemulsion at a ICG identical dose of 2 mg/kg, respectively. At 24 h post-injection, the blood samples were collected and followed by sacrificing the mice. The blood samples were used to detect the RBC, PLT, WBC, HGB, ALT, AST, BUN, and CREA parameters. The major organs, including heart, liver, spleen, lung, kidney, were dissected for H&E staining.

## Results and Discussion

**FIGURE 1:**
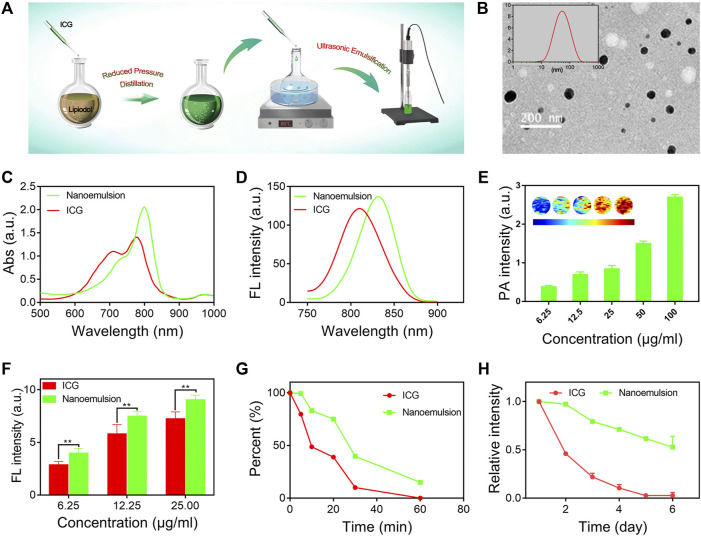
**(A)** Schematic illustration of the preparation process of nanoemulsion. **(B)** TEM image and size distribution of nanoemulsion. **(C)** NIR-UV−vis spectra and **(D)** FL spectra of free ICG solution and nanoemulsion. **(E)** PA imaging signals of nanoemulsion with different ICG concentrations. **(F)** FL intensity comparison of free ICG solution and nanoemulsion at different concentrations. **(G)** FL intensity change of ICG and nanoemulsion after laser (2 W/cm^2^, 808 nm) irradiation. **(H)** FL intensity change of ICG and nanoemulsion after different storage times.

The ICG-lipiodol nanoemulsion was prepared *via* a self-emulsifying method with proper modification (Figure 1A), and further characterized with morphology, optical performances, and stability. As observed from the transmission electron microscopy (TEM) image shown in Figure 1B, the developed nanoemulsion had a well-defined spherical structure with a narrow size distribution. The hydrodynamic diameter and ζ-potential of nanoemulsion was about 60 nm and -5 mV by the dynamic light scattering (DLS) analysis (Figure 1B insertion). Compared to free ICG solution, the nanoemulsion showed obvious enhanced and red-shifted absorption with a maximum absorption peak of 805 nm (Figure 1C). Similarly, a significant red-shift of fluorescence emission spectra between ICG (810 nm) and nanoemulsion (840 nm) was also observed (Figure 1D). Such shifted absorption and emission peaks may be attributed to the different external mediums of ICG that the nanoemulsion disperses ICG molecules in oily lipiodol instead of direct dispersion in water like free ICG solution. When imaging *in vivo*, the red-shifted optical performances of nanoemulsion are conducive to overcome the tissue absorption and scattering, implementing accurate intraoperative navigation. In addition to fluorescence imaging, the potential ability of nanoemulsion as a photoacoustic (PA) imaging and computed tomography (CT) imaging contrast agent was also evaluated. As expected, the PA and CT signals steadily enhanced with the concentration of nanoemulsion increasing, which can be clearly visualized in photographs (Figures 1E, Supplementary Figure S1).

Fluorescence performance of contrast agent is an important factor governing the outcomes of intraoperative imaging. Successful surgical resection of HCC closely relies on strong FL intensity, great anti-photobleaching ability, and excellent FL stability (Nagaya et al., 2017). In this study, we systemically investigated the FL performance of prepared nanoemulsion. It was found that nano-emulsification did not disrupt the FL characteristics of ICG (Figure 1F). On the contrary, the FL intensity of ICG from nanoemulsion was remarkably higher than that from free ICG solution, which suggests great potential in improving SNR of intraoperative imaging. Upon laser irradiation (808 nm, 2 W/cm^2^, 10 min), the ICG solution reduced its FL intensity to almost 50%, while 80% of FL intensity was remained in the group of nanoemulsion (Figure 1G). Moreover, after 60 min light exposure, the nanoemulsion still exhibited FL imaging performance, but the ICG solution has completely photobleached without any FL signal. Such potent anti-photobleaching ability is expected to prolong the working period of ICG-mediated FL imaging, as a result, the time window of surgical navigation promisingly extends. In clinical practice, the ICG solution should be freshly prepared when using due to its self-quenching phenomenon. However, in our study, the ICG molecules after nano-emulsification showed highly enhanced FL stability (Figure 1H). Different from ICG solution whose FL signal rapidly declined to zero on the fifth day, the FL intensity of nanoemulsion stored for 6 days was still more than 50%. The great FL stability can be explained by the strong viscosity of lipiodol, which significantly restricts the movement of ICG molecules and therefore reduces molecular packing-induced FL quenching (Zweck and Penzkofer, 2001). Further morphology stability evaluation also demonstrated the great stability of nanoemulsion (Supplementary Figure S2). Its diameter stayed pretty constant during 2 months. Benefiting from the red-shifted optical spectra, superior fluorescence performances, and enhanced stability, the nanoemulsion could serve as a novel-acting contrast agent for FL-guided intraoperative imaging.

**FIGURE 2:**
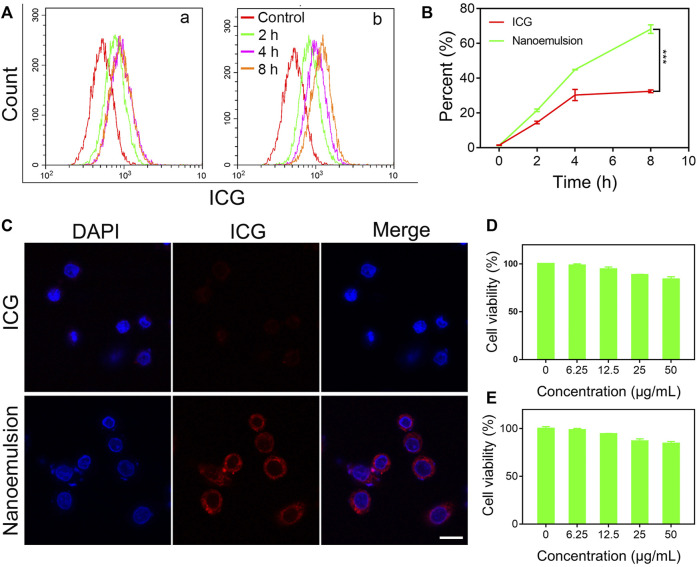
**(A)** Flow cytometry profiles, **(B)** quantitative analysis, and **(C)** CLSM images of ICG and nanoemulsion internalization by HepG2 cells. Cell viability of **(D)** LO2 cells and **(E)** HepG2 cells treated with nanoemulsion at different ICG concentrations.

Efficient uptake by tumor cells is the fundamental step for contrast agent in the optical surgical navigation and can lead to a more precise and effective HCC resection. To validate the internalization ability of nanoemulsion in the cellular environment, the human hepatoma cell line (HepG2) was selected. As shown in Figures 2A,B, both ICG solution and nanoemulsion exhibited a time-dependent internalization by HepG2 cells. With increasing co-incubation time, the cellular uptake of ICG became enhanced. Comparatively, the ICG after nano-emulsification performed dramatically higher cellular internalization efficiency than its free state. After 8 h co-incubation, the uptake rate of nanoemulsion was 68.23%, more than twice that of pristine ICG solution (32.44%). This result was also supported by confocal laser scanning microscopy (CLSM) assay in which the nanoemulsion-treated HepG2 cells presented bright red fluorescence in cytoplasm, but the red ICG signal was almost invisible in the cells incubated with ICG solution (Figure 2C). Such efficient cellular uptake of nanoemulsion may be attributed to the combination of good cell membrane affinity provided by lecithin carrier and deposition effect of lipiodol. For future biomedical applications, toxicity testing of nanoemulsion is a critical requirement. Its cytotoxicity was studied in a human hepatic cell line (LO2) and HCC line (HepG2). MTT assays showed no significant toxic effect on the two cell lines after incubation with nanoemulsion for 24 h (Figures 2D,E). When the ICG concentration from nanoemulsion is up to 50 μg/ml, the viability of both cell lines still maintained higher than 80%. This good cellular safety, coupled with efficient tumor cell uptake, greatly supports the bio-application of nanoemulsion for intraoperative imaging of HCC.

**FIGURE 3:**
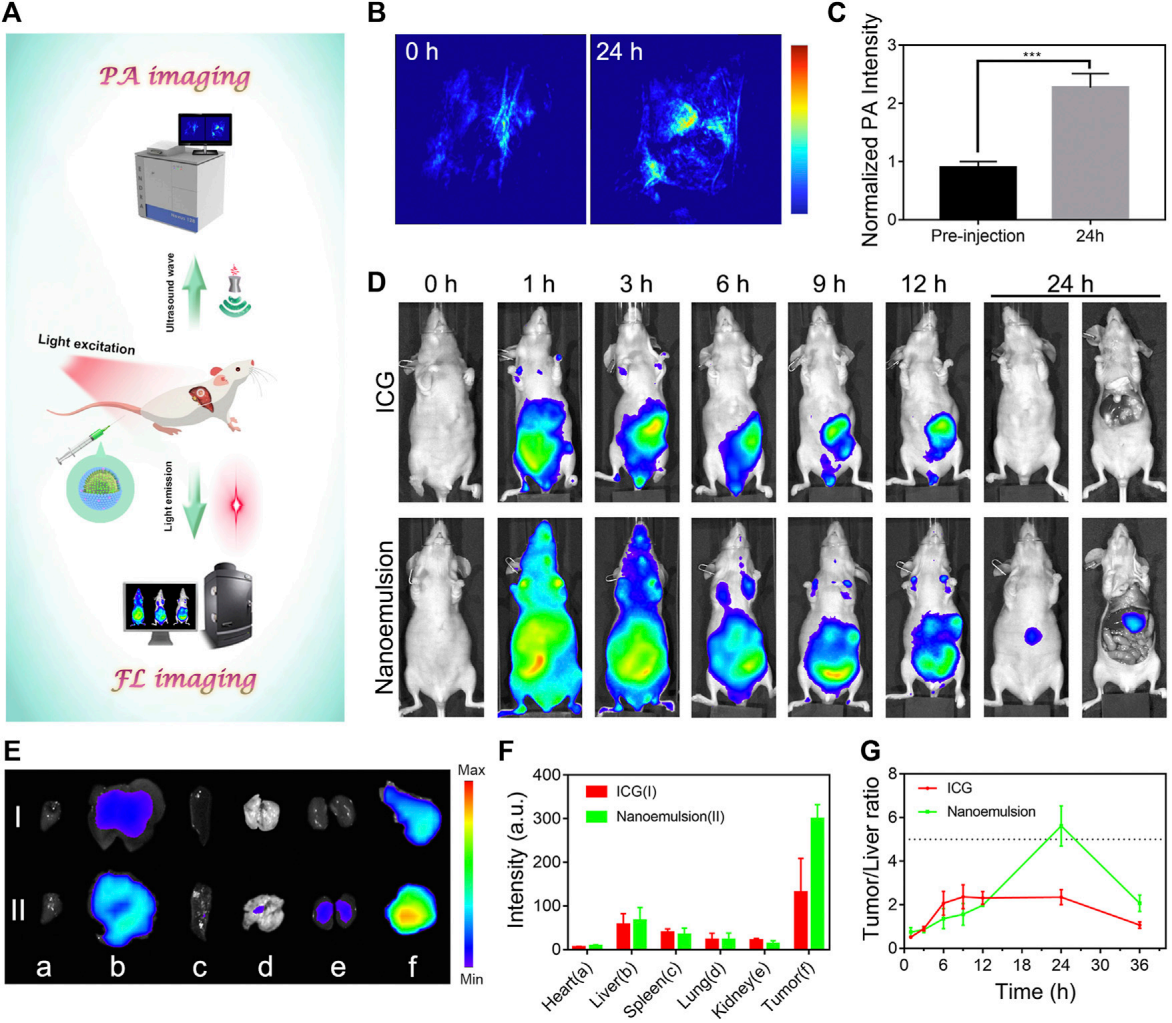
**(A)** Schematic illustration of the optical diagnosis of nanoemulsion in orthotopic HCC-bearing mice. **(B)** PA images and **(C)** intensity analysis of tumor sites from nanoemulsion-treated mice. **(D)** FL images of orthotopic HCC-bearing mice model after tail vein injection of ICG solution or nanoemulsion. **(E)** *Ex vivo* tissue FL image and **(F)** FL intensity (I: ICG group; II: nanoemulsion group) of HCC-bearing mice after different treatments at 24 h post-injection. The a, b, c, d, e, and f represent heart, liver, spleen, lung, kidney, and tumor. **(G)** FL signal ratio of tumor-to normal liver tissue at various time points.

Accurate diagnosis of HCC is imperative to guide surgical resection and control the metastasis of this disease (Alam et al., 2018; Pang et al., 2020). Considering the unique imaging properties of ICG (Chu et al., 2020), the nanoemulsion is expected as a powerful contrast agent to reveal the tumor margins. We evaluated multi-modality cancer visualization by prepared nanoemulsion in orthotopic HCC-bearing mice (Figure 3A). The PA images were acquired before and after intravenous injection of nanoemulsion for 24 h. As shown in Figures 3B,C, the PA signal was notably stronger in the tumor than in healthy hepatic tissue, indicating the great potential of nanoemulsion in HCC PA imaging *in vivo*. To verify the *in vivo* targeting ability of nanoemulsion and the optimum time point for intraoperative imaging, we then compared and analyzed the FL imaging results at different time points after administration. Figure 3D shows that free ICG was rapidly accumulated to the abdomen within 1 h. No obvious FL signal from liver region was detected during the entire monitoring period. However, after being packaged into nanoemulsion, the ICG showed a different biodistribution behavior, with gradual FL attenuation in normal sites but a time-dependent FL enhancement at the liver tissue. At 24 h post-injection, the FL signal was completely cleared from abdomen, only concentrating to the liver area. Subsequently, the main organs and tumor of mice were isolated for distribution analysis (Figures 3E,F). Obviously, mice treated with free ICG solution exhibited weak FL signal intensity, suggesting the fast metabolism and clearance of ICG from body. By contrast, the FL signal from nanoemulsion-treated mice was only weak in the organs, but was rather strong in the tumor tissue. The higher FL signal of nanoemulsion can be explained by the enhanced FL intensity and stability of ICG after nano-emulsification, and the efficient tumor accumulation is attributed to the tumor-associated EPR effect. Figure 3G shows the tumor-to-normal liver tissue ratio of the FL signal at different time points. It can be seen that the maximal tumor signal over liver contrast was only 2-fold at 6 h post-injection of free ICG solution, which is nearly one-third of that from nanoemulsion-treated mice (5.7-fold at 24 h post-injection). Such significant positive contrast enhancement in the tumor site, but gradually reduced signal in healthy liver tissue, greatly ensures a clear visualization of tumor margin. Observed by the FL and bioluminescence imaging (Supplementary Figure S3), the resected tumor by nanoemulsion-mediated FL guidance was exactly coincident with the tumor formed by luciferase-transfected HepG2 cells, revealing the potent ability of nanoemulsion in pinpointing tumor margin.

**FIGURE 4:**
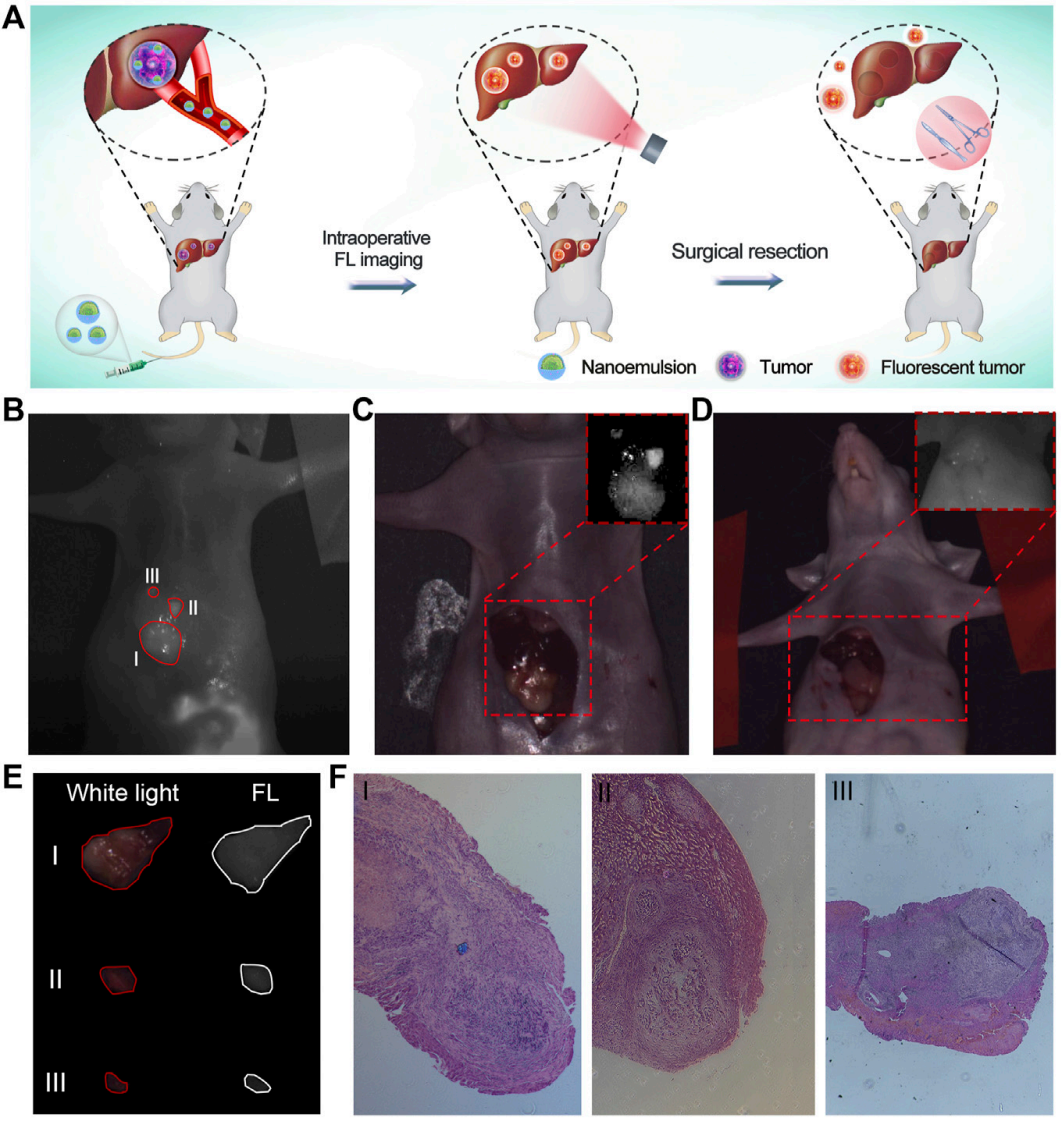
**(A)** Schematic illustration of nanoemulsion for FL imaging-guided surgical resection of HCC. **(B)** Post-mortem FL images of orthotopic HCC-bearing mice under surgical navigation imaging system. Image was taken 24 h after injection of nanoemulsion. I, II, and III represent tumors with different sizes. **(C)** Pre-operative and **(D)** Post-operative white light images of orthotopic HCC-bearing mice. Insertions are the FL images of liver region under surgical navigation imaging system. **(E)** White light and FL images of resected tissues under the nanoemulsion-mediated FL guidance. **(F)** Histological analysis of tumor specimens from the resected tissues.

After verifying the maximal tumor-to-liver tissue ratio of nanoemulsion, orthotopic hepatic tumor-bearing mice (*n* = 3) were subjected to laparotomy with general anesthesia and subsequent luminescence imaging liver-resection guidance at 24 h post-injection of nanoemulsion (Figure 4A). With the guidance of nanoemulsion-mediated FL imaging, some tissues in the liver region showed bright fluorescence signal and then were removed (Figures 4B–D). Strikingly, the FL signal at the resection area disappeared after the surgery (Figure 4D), which could provide a timely feedback to the surgeon regarding the surgical outcome. Under the guidance of nanoemulsion-mediated FL imaging, the delineated tissues were resected (Figure 4E). Postoperative histological analysis of the excised tissues showed disordered structure and mass tumor cell infiltrating (Figure 4F), suggesting that the tumor tissues, even small volumes, could be illuminated and removed completely. Further safety examination revealed that the nanoemulsion was nontoxic to organs (Supplementary Figure S4), and showed negligible changes in peripheral blood (Supplementary Figure S5), liver functions (alanine transaminase and aspartate transaminase), and renal functions (creatinine and blood urea nitrogen) (Supplementary Figure S6). All of these demonstrated that the developed nanoemulsion could be competent for an effective and safe platform to accurately highlight tumor lesions, guiding HCC intraoperative resection.

## Conclusion

In this study, we reported a novel-acting nanoemulsion for NIR FL imaging-guided HCC surgery. This highly potent nanoemulsion was prepared using self-emulsifying nanotechnology to facilely bridge ICG and lipiodol, and shows three primary advantages as follows: 1) superior FL performances, including red-shifted optical spectra, enhanced FL intensity, great anti-photobleaching ability and excellent FL stability; 2) distinguished HCC targeting and accumulation, achieving an ultrahigh tumor-to-liver FL signal ratio of 5.7 for precise surgical navigation; 3) great biosafety that all of the materials prepared for nanoemulsion are clinically approved. Under the guidance of nanoemulsion-mediated NIR FL imaging, the tumor margins were clearly delineated and resected from orthotopic HCC-bearing mice. Such fluorescent nanoemulsion could represent a promising real-time imaging platform to improve clinical intraoperative quality, reduce the risk of postoperative complications, and even potentially shift the current paradigm of optical surgical navigation.

## Data Availability

The original contributions presented in the study are included in the article/Supplementary Material, further inquiries can be directed to the corresponding authors.
