# Supplementary material for: Superior Fluorescent Nanoemulsion Illuminates Hepatocellular Carcinoma for Surgical Navigation
Source: Front Bioeng Biotechnol. 2022 Apr 25;10:890668. doi: 10.3389/fbioe.2022.890668 (PMC9081524; doi:10.3389/fbioe.2022.890668)
Supplement: Supplementary file 1 [file DataSheet1.PDF]

Supplementary Material

Supplementary Material

1 Supplementary Figures

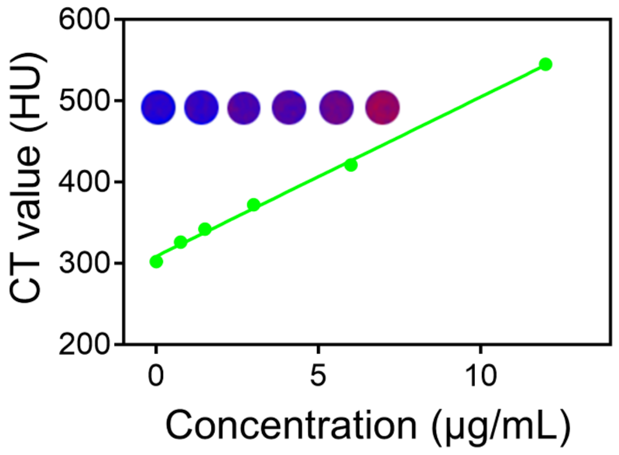

Figure S1. CT images and value of nanoemulsion at different iodine concentration.

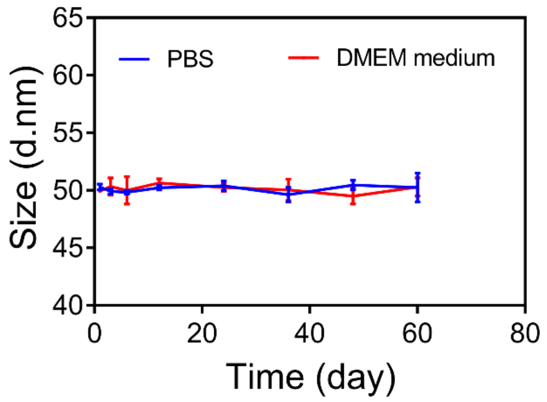

Figure S2. Hydrodynamic diameter of nanoemulsion in PBS and DMEM medium at different times.

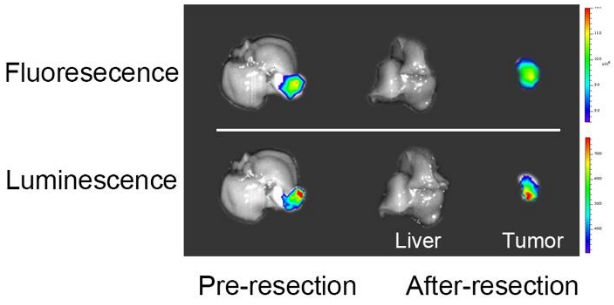

Figure S3. Fluorescence imaging and luminescence imaging of tumor-bearing liver after i.v. injection nanoemulsion.

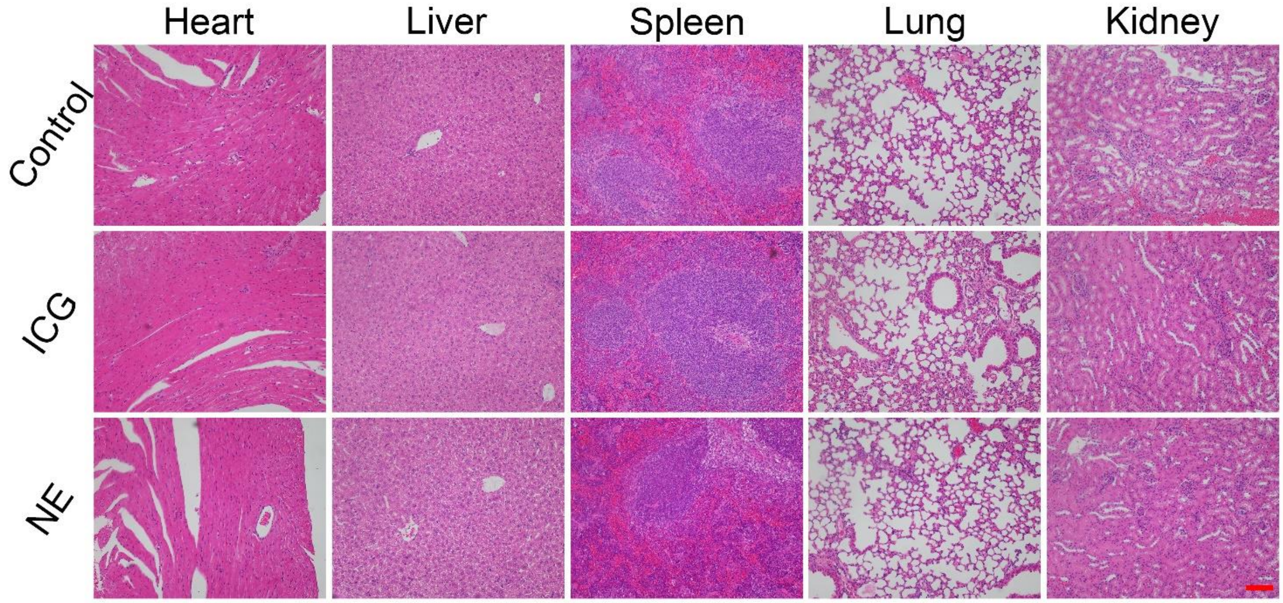

Figure S4. Histopathologic examination of the tissues including heart, liver, spleen, lung and kidney from BALB/c nude mice after intravenous injection different agents. (Scale bar =100 μm).

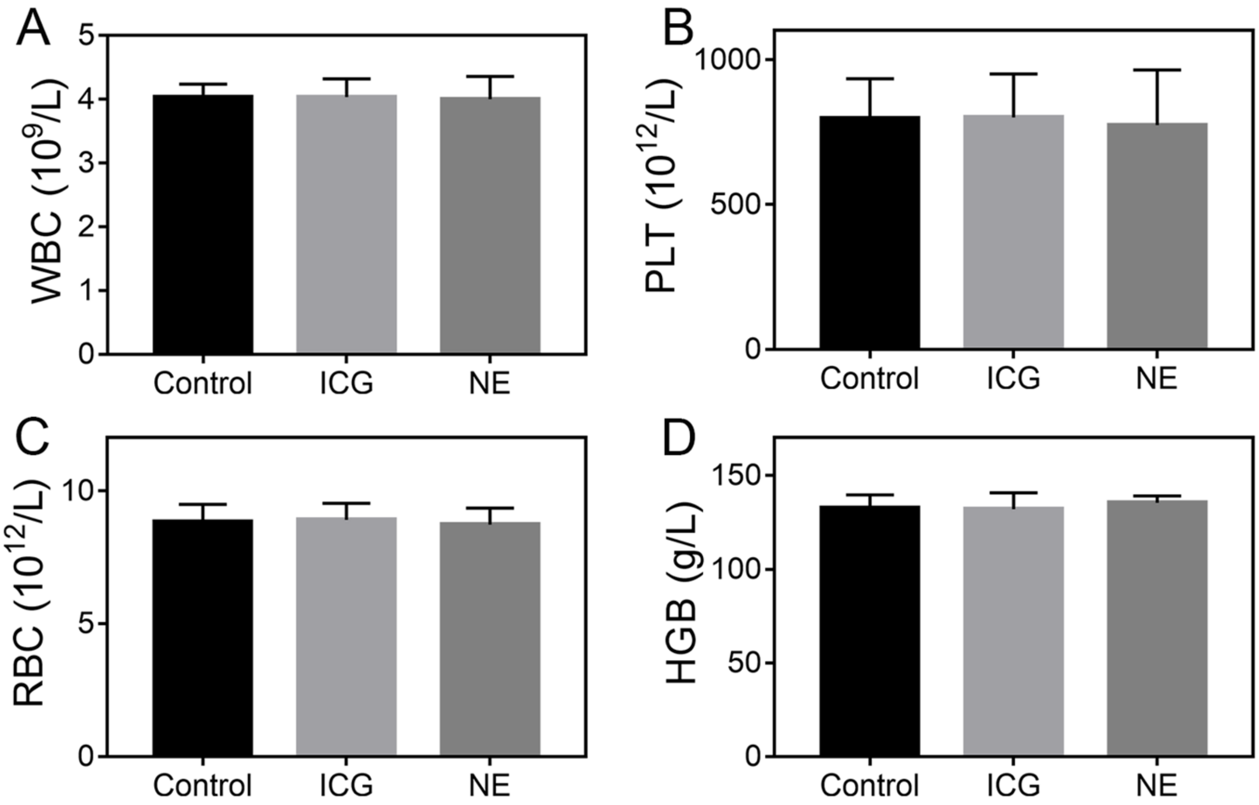

Figure S5. The hematological parameters of nude mouse after i.v. injection saline, ICG and nanoemulsion.

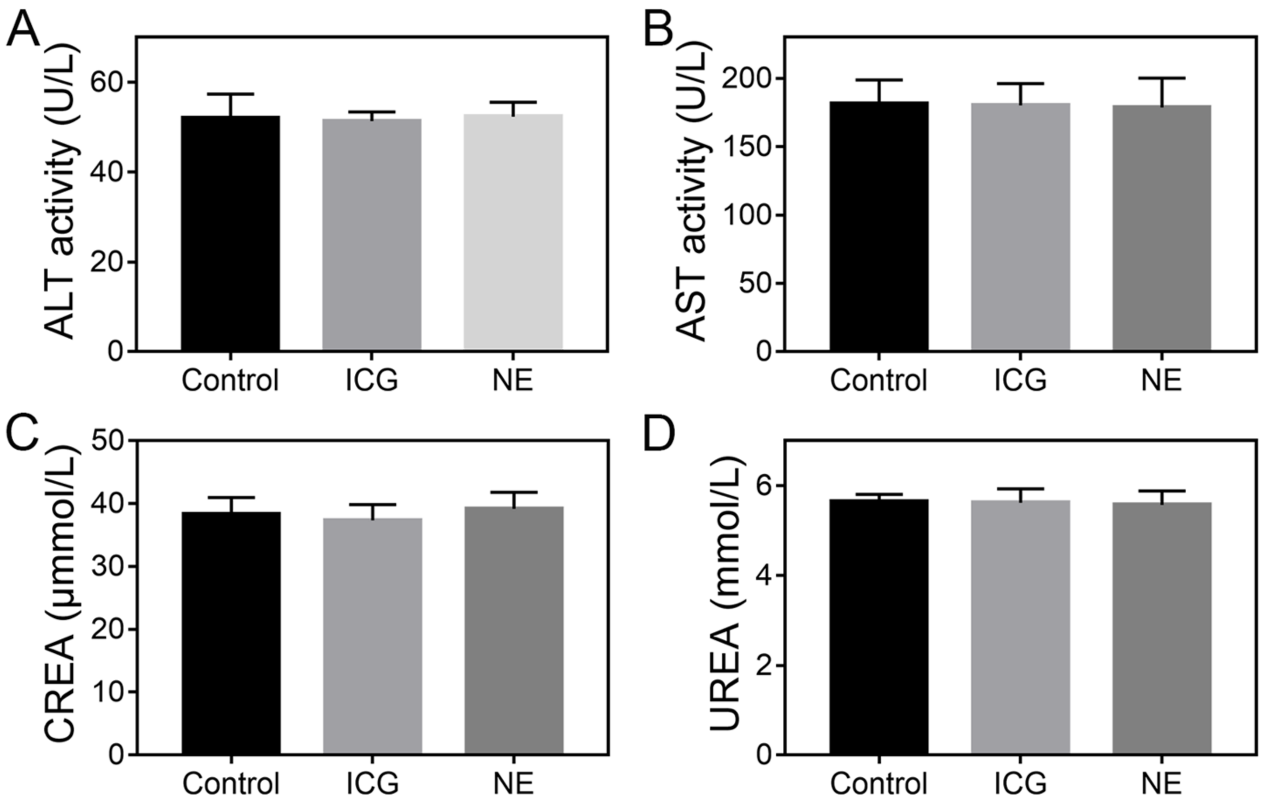

Figure S6. Serum alanine aminotransferase (ALT), aspartate aminotransferase (AST), blood urea (UREA) and creatinine (CREA) of mice after intravenous injection different agents.
